# Spatial Association Network Evolution and Variance Decomposition of Economic Sustainability Development Efficiency in China

**DOI:** 10.3390/ijerph20042966

**Published:** 2023-02-08

**Authors:** Xin Fang, Yun Cao

**Affiliations:** 1 School of Business, Macau University of Science and Technology, Macau 999078, China; 2 School of Economics, Ocean University of China, Qingdao 266100, China; 3 Marine Development Studies Institute of OUC, Key Research Institute of Humanities and Social Sciences at Universities, Ministry of Education, Qingdao 266100, China

**Keywords:** sustainable economic development efficiency, EBM-Malmquist model, social network analysis

## Abstract

The economy’s sustainable development has become a national strategic deployment in China. Research on the difference between the economic sustainable development efficiency (ESDE) and the spatial network will assist the government with the deployment of sustainable development strategies and the achievement of the “peak carbon dioxide emissions”. This paper designs the input–output indicator system of sustainable economic development efficiency and builds an unexpected output super-EBM-Malmquist model to measure the ESDE of 30 provinces in China from 2008–2020. According to the ranking of ESDE, the 30 provinces in China are classified into four groups by applying the quartile method, and the difference in the ESDE in different regions and the temporal variation of different provinces are studied by using the Dagum Gini coefficient and Gaussian Kernel density. Moreover, the relationship between ESDE in different provinces is studied based on the revised gravity model and social network analysis method. The connections between provinces with related relations constitute the ESDE network. Results show that (1) the average ESDE in China shows an upward trend, the eastern region is in a leading position, the central and western regions are trying to catch up with the eastern region, and the development of the northeast region is lagging behind. (2) The level of ESDE in different provinces is clearly arranged from high to low, illuminating a distinct pattern. Moreover, provinces with high levels of development are much higher than provinces with low levels of development, presenting a phenomenon of polarization. (3) The regional ESDE development imbalance is prominent, and the ESDE in the eastern region is closely related, while the connection in the western region is lower. (4) Beijing–Tianjin Urban Agglomeration and the Yangtze River Delta have significant spatial spillover effects in the association network, while the northeast, northwest, southwest and central regions have significant spatial benefit relationships. These findings provide important enlightenment for promoting the sustainable and balanced development of China’s economy.

## 1. Introduction

With the growing energy consumption, energy demand and energy use to promote economic growth at the expense of environmental sustainability [1,2], sustainable economic development is facing a huge threat. As the world’s second largest economy, China’s energy consumption structure has long been dominated by fossil energy, its economic model is characterized by significantly high consumption and high emissions, and its rapid economic growth is accompanied by environmental pollution and ecological degradation [3].

China’s economic development has been maintaining the characteristics of crude growth with high input and high consumption and low output, and the energy structure dominated by coal and high overall carbon emissions is the reality of China’s national situation. According to BP statistics, coal will account for 56% of total energy consumption in 2021, and China has become the world’s largest emitter of carbon dioxide. Obviously, this energy structure is unsustainable and will cause irreversible damage to the ecological environment [4]. The high consumption of primary energy, especially the large-scale exploitation of fossil energy, has led to an excessive amount of carbon dioxide emissions and increasingly serious environmental impacts, which is contrary to the principles of sustainable development [5,6,7]. Sustainable economic development is becoming a major concern for governments [8], especially after a significant increase in the exploitation of natural resources that has led to serious environmental problems [9]. In the 1970s, the economist Meadows argued in *The Limits to Growth* that technological progress plays a crucial role in the harmonization of economic development with resources and the environment. In the context of tightening resources and environmental constraints, “carbon peaking”, “carbon neutrality” and “emission reduction”, rapid economic development cannot be achieved at the expense of resources and the environment. Therefore, the sustainable development of China’s economy necessarily needs to be discussed within the framework of environmental protection [10], and the study of economic sustainability under resource and environmental constraints has gradually become the focus of academic attention.

The Chinese government gives great importance to sustainable economic development. How to reconcile economic development with the environment and how to enhance the level of sustainable economic development in the future are key issues for sustainable economic development [11,12]. In 2015, China proposed supply-side structural reform, which aims to restructure the economy, achieve an optimal allocation of factors, improve the quality of economic growth and promote sustainable economic development. In the report of the 19th National Congress, General Secretary Xi Jinping emphasized the development concept of “green water and golden mountains are golden mountains and silver mountains”, highlighting the importance that the government attaches to the organic integration of economic development and ecological protection. In the 14th Five-Year Plan of China, the importance of sustainable economic development was re-emphasized. In the framework of environmental protection and energy constraints, it is therefore of great theoretical and practical importance to discuss the sustainable development of China’s economy.

This study measures the ESDE in China with the aim of identifying gaps in the ESDE across different regions and provinces while also examining the spatial network structure of sustainable economic development to help the government implement environmental protection, conservation and economic sustainable development strategies, and to raise China’s level of sustainable development. This study designs a research framework model of “efficiency measurement—differentiation analysis—network structure”. Gini coefficient and Kernel density estimation are two tools for the difference measurement of an object among different individuals, and they also provide a good visualization. In particular, the Gini coefficient method is able to calculate the development gap among the eastern, western and central regions of China, and can decompose the existing gap to distinguish that the difference in the sustainable development level of the national economy mainly comes from the gap among the three major regions or the gap within the three major regions. Moreover, Kernel density estimation is also a method to analyze differences and gaps in economic sustainability and result visualization. The social network analysis method is a method that can visualize the flow relationship of sustainable economic development among different provinces. It clarifies the closeness of the level of sustainable economic development in different regions, thus providing information for governments to take further actions that are tailored to local conditions and better promote China’s sustainable economic development. Furthermore, in addition to estimating the ESDE, this study applies ESDE outcomes to further measure the regional development gap, the regional development evolution pattern, and the spatial network structure.

## 2. Literature Review

### 2.1. Defining Sustainable Economic Development

Sustainable development is one of the most common words used today and represents an expression to stop environmental degradation, which can lead to ongoing climate change and changes in citizens’ lifestyles and habits [13]. The definition, connotation and characteristics of sustainable development have been defined in the economics literature for a long time, and although there is no unified and clear theoretical framework, it has had a significant impact [14]. Stivers [15] defines the sustainability of economic growth as “an economy in equilibrium with its ecological support system”. The concept of “sustainable development” was first introduced in 1987 in the report of the United Nations World Commission on Environment and Development on the *Future of Humanity: Our Common Future*. Pointing to the need to change the development paradigm for the benefit of present and future generations, the report defines sustainable development as development that meets the needs of the present without compromising the ability of future generations to meet their needs [16], and this definition is widely used by international organizations and relevant scholars. Pezzey [17] argues that the core meaning of sustainable development is a development path that keeps the welfare of the population from declining, and Dailami, et al. [18] believes that sustainable economic growth can be an important element of the pace of economic development; Barro [19] defines sustainable economic growth as encompassing characteristics such as gross domestic product, the political system, income distribution, health status and religious beliefs. Brock and Taylor [20] proposes that sustainable economic growth is primarily about economic growth and environmental quality improvement. In 2005, the World Summit for Social Development adopted the ‘3 E’s’ of economic, equity and environmental as the core elements of sustainable development. The UN Sustainable Development Goals (SDGs) aim to address the social, economic and environmental dimensions of development in an integrated manner between 2015 and 2030, and to shift towards a sustainable development path. Many other scholars have defined and discussed various aspects of sustainable development, including its definition, connotation and applicability, from the perspectives of ecology and environmental economics [21,22].

### 2.2. Factors Influencing Sustainable Economic Development

Scholars have studied the different influencing factors of economic sustainability because of its rich connotations. Wang and Huang [23] found through bibliometric research methods that research on sustainable development in the context of COVID-19 is broad in scope and covers a wide range of disciplines, but it is lacking in depth overall. While developed countries are working on the sustainability of education, developing countries are more concerned with economic sustainability.

Khan, et al. [24] used the Environmental Kuznets Curve (EKC) to explore the non-linear link between energy intensity, financial development and environmental sustainability, showing a non-linear inverted ‘U’ shaped relationship between financial development and environmental sustainability. Bai, et al.’s [25] study of China’s economic sustainability goals is based on a panel threshold model. The study finds that investing in renewable energy reduces greenhouse gas emissions, thereby maintaining sustainable economic growth and contributing to the achievement of the SDGs. Dabbous and Tarhini [26] applied a fixed effects panel model to find that a sharing economy contributes to sustainable economic development. Hosan, et al. [27] used a panel model to analyze the links between the demographic dividend, digital innovation, energy intensity and sustainable economic growth in 30 emerging economies from 1995 to 2018, finding that the demographic dividend and digitalization contributed to sustainable economic growth, while energy intensity was negatively associated with sustainable economic growth.

### 2.3. Economic Sustainable Development Efficiency (ESDE)

Economic sustainability is a multidisciplinary subject of study in all the countries of the world, and it is critical to any country’s economic development and environmental protection to assess a country’s sustainable economic development performance and outcomes [28]. In terms of the selection of models for measuring efficiency, a number of scholars have chosen traditional radial DEA models or non-radial SBM models. Studies mostly quantify GDP as the desired output and environmental pollutants as the non-desired output, and establish a system of input–output indicators. DEA (Data Envelopment Analysis) models that can handle multiple inputs and outputs are the common tools used. Qu, et al. [29], constructed an improved super-efficient radial network DEA model to assess China’s regional sustainable development performance from 2009 to 2017; the study shows that there are significant differences in the level of sustainable development in different regions, with the eastern region having the highest level of sustainable development and the central and western regions being in a steady catch-up stage of development. Many scholars consider undesired output as an output variable and construct a non-radial SBM model (Slacks-Based Measure), which can handle efficiency measurements with undesired output variables. In recent years, the SBM model has been widely used in the evaluation of green economic efficiency [30,31]. Wen, et al. [32] used a three-stage SBM model to measure the sustainable development efficiency of counties in the Yellow River Basin from 2005 to 2015, and they classified the efficiency of different regions into low, medium-low, medium-high and high-efficiency through quadratic plots, revealing the spatial clustering pattern of sustainable development. Swain and Ranganathan (2021) [33] employed a network perspective to analyze IAEG-SDG data on SDG indicators from the UN Sustainable Development dataset for the period 2000–2017, finding that the same criteria cannot be set for sustainable development goals in different regions and that different priority development goals should be set for different SDG groups. Wang and Jia [34] applied the DEA-SBM super efficiency model to characterize sustainable economic growth, demonstrating that improving the energy consumption mix and controlling CO_2_ emissions both contribute to sustainable economic growth.

However, neither the traditional radial DEA model nor the non-radial SBM model is capable of dealing with the situation where the input and output variables have both radial and non-radial characteristics, and the information on the ratio between the target and actual values of inputs or outputs is easily lost, resulting in an overestimation or underestimation of the efficiency values [35]. EBM has been proposed as a hybrid distance model to overcome the shortcomings of the DEA and SBM models and to improve the accuracy of the evaluation results [36]. However, there are not many cases where the EBM model has been applied to evaluate the ESDE.

### 2.4. Spatial Variation and Social Network Analysis

In research fields as efficiency evaluation, scholars are concerned not only with overall efficiency, but also with the analysis of the differences in efficiency and the spatial structure of networks [37,38]. The analysis of spatial differences is one of the key issues in geographic studies, allowing for a precise portrayal of the spatial differences in the study population, and thus the implementation of policies corresponding to regional differences [39]. Currently, a number of studies have adopted methods such as the Gini coefficient [40,41], spatial exploratory data analysis [42] and Kernel density functions [43] to analyze spatial differences under different spaces. With the advancement of China’s coordinated regional development strategy and sustainable development strategy, the spatial linkages between regions are increasingly taking the form of complex networks [44]. It is hardly enough to analyze the ESDE by relying only on the variance analysis of “attribute data”. Economic efficiency issues are often characterized by the evolution of spatially linked network structures, which requires the use of social network analysis [38,44].

Social network analysis is a method based on relational data to study the complexity of the network structure between unit nodes, effectively overcoming the shortcomings of “attribute data”, and has been widely used in recent years in the study of complex correlation networks in the fields of regional energy and environment [45,46]. Scholars have combined variance analysis and social network analysis to analyze the dynamic evolution of spatial differences in efficiency issues. Shen, et al. [47] applied the Theil index and Moran indices to assess the characteristics of carbon emissions in the Yangtze River Delta urban agglomeration, the Chengdu-Chongqing urban agglomeration and the Guangdong-Hong Kong-Macao urban agglomeration, combined with the social network analysis method to examine the network structure characteristics of the three urban agglomerations from three perspectives: overall, individual and correlation. Based on the impact of the spatial correlation of carbon emissions on sustainable economic development, Zhang, Tai, Cheng, Zhu and Hou [46] constructed a carbon emission efficiency network and classified carbon emission efficiency into five spatial classes: low efficiency class, low efficiency class, medium efficiency class, high efficiency class and higher efficiency level and analyzed their spatial differences and dynamic evolution. However, there is less literature on the application of the Gini coefficient, Kernel density estimation and social network analysis as holistic research methods to the research topic of economic sustainable development efficiency. Although a few studies have also verified and discussed the spatial agglomeration characteristics and spillover effects of sustainable development based on geographical proximity or economic proximity, the current research on the structural characteristics of spatially linked networks of sustainable economic development efficiency and their spatial dynamic differences still needs to be deepened.

The contribution of this study lies in several areas. Firstly, this research extends the study of sustainable economic development to cover the level of efficient use. We take 30 provinces in China as the study objects and classify the sustainable development efficiency levels into very high efficiency, high efficiency, medium efficiency and low efficiency in order to reveal the regional spatial and temporal characteristics and the mechanisms of differences, and to better analyze the spatial evolution characteristics of China’s sustainable economic development efficiency and help to achieve sustainable and efficient economic development in China. Secondly, this study provides an accurate estimate for the measurement of sustainable economic development efficiency by using an over-efficiency EBM model of non-expected output, which takes into account the non-expected output of sustainable economic development. Thirdly, this study uses the Gini coefficient, Kernel density estimation and social network analysis as a framework model for the study of spatial dynamic differences to analyze the spatial structure and evolutionary characteristics of the efficiency of sustainable economic development from the perspective of spatial correlations.

## 3. Methods and Data

### 3.1. Index System and Data Sources

The core connotation of sustainable economic development is to organically integrate the environment, resources and development issues. It not only pays attention to the rapid economic growth, but also needs to pay attention to the quality of economic development. We emphasize that sustainable economic development cannot degrade the quality of the environment or harm natural resources at the cost of being sacrificed. Sustainable economic development ensures maximum economic development on the premise of maintaining the quality of natural resources and maintaining environmental stability.

Most of the literature is an economic sustainable growth index system that incorporates labor, capital, technology and energy consumption into the input indicators of production factors [48] but does not take into account the input of environmental protection and social livelihood. However, the ESDE is based on the connotation of sustainable economic development, which needs to fully consider the comprehensive economic efficiency after the cost of the environment and resources. This paper draws on the existing evaluation system based on the principles of system, science and data availability, considering the constraints of resources and the environment. On this basis, this paper designs six dimensions of input indicators, including environment, resources, social livelihood, technology, capital and labor, as well as output indicators such as expected output and undesired output, to construct an evaluation index system for the ESDE.

Considering data availability and continuity, this paper selects 30 provincial-level administrative regions in China (excluding Hong Kong, Macao, Taiwan and Tibet) as the research unit, and takes 2000–2020 as the research period. The data come from the National Bureau of Statistics, “China Statistical Yearbook”, “China Population and Employment Statistical Yearbook”, “China Energy Statistical Yearbook”, “China Environmental Statistical Yearbook”, “China Science and Technology Statistical Yearbook” and provincial statistical yearbooks. The specific index system is shown in Table 1. Among them, the capital input index is represented by the stock of fixed asset investments. Because the EBM model limits the number of indicators, this paper uses the entropy method to calculate the weight of multiple secondary indicator variables. The entropy weight method is an objective weighting method for indicators, which can deeply reflect the distinguishing ability of indicators. Compared to the subjective weighting method, the outcome of entropy weighting method is more reliable. Finally, input indicators such as capital, environmental governance, social security, technology, resources and labor are obtained, with GDP as the final expected output and COD and SO_2_ as undesired outputs. The specific indicators are shown in Table 1.

### 3.2. Super-EBM Model of Unexpected Output

#### 3.2.1. Super-EBM Model

The effective unit efficiency value measured by the EBM model is 1; it is difficult to further analyze the efficiency difference of the effective evaluation unit, and the efficiency of different evaluation units cannot be judged. Andersen and Petersen [49] put forward the idea of super-efficiency planning borrowed from the design idea of Tone’s [50] super- SBM model, which optimized the constraints of the EBM model with no guidance and unexpected output, and the efficiency measurement result was equal to 1 unit for secondary calculation. The formula of the super-efficient EBM model is as follows [51].

The constraints on input variables are

(1) $$\sum_{j=1,j\neq k}^{n}\lambda_{j}x_{ij}-s_{i}^{-}\leq \theta x_{ik}\;i=1,2,\cdots ,m$$

The constraints on the expected output factors are

(2) $$\sum_{j=1,j\neq k}^{n}\lambda_{j}y_{rj}+s_{r}^{+}\geq \varphi y_{rk}\;r=1,2,\cdots ,s$$

The constraints on the unexpected output factors are

(3) $$\sum_{j=1,j\neq k}^{n}\lambda_{j}b_{pj}-s_{p}^{b-}\leq \varphi b_{pk}\;p=1,2,\cdots ,q$$

The objective function is

(4) $$\gamma^{*}=\min\frac{\theta +\varepsilon_{X}\sum_{i=1}^{m}\frac{w_{i}^{-}s_{i}^{-}}{x_{ik}}}{\varphi -\varepsilon_{y}\sum_{r=1}^{s}\frac{w_{r}^{+}s_{r}^{+}}{y_{rk}}+\varepsilon_{b}\sum_{p=1}^{q}\frac{w_{p}^{b-}s_{p}^{b-}}{b_{pk}}}$$

The constraints of the super-EBM model are

(5) $$s.t\left\{ \begin{array}{l} \sum_{j=1,j\neq k}^{n}\lambda_{j}x_{ij}-s_{i}^{-}\leq \theta x_{ik}\;i=1,2,\cdots ,m\\ \sum_{j=1,j\neq k}^{n}\lambda_{j}y_{rj}+s_{r}^{+}\geq \varphi y_{rk}\;r=1,2,\cdots ,s\\ \sum_{j=1,j\neq k}^{n}\lambda_{j}b_{pj}-s_{p}^{b-}\leq \varphi b_{pk}\;p=1,2,\cdots ,q\\ \lambda_{j}\geq 0,s_{i}^{-}\geq 0,s_{r}^{+}\geq 0,s_{p}^{b-}\geq 0 \end{array}\right.$$

where $x_{ij}$ is the data matrix of input factor indicators, and $y_{rj}$ is the data matrix of output factor indicators. Variable $\gamma^{*}$ is the best efficiency with constant returns; $s_{r}^{+}$ is the slack variable of expected output of category r; $s_{p}^{b-}$ is the slack variable of the unexpected output of type  p; $w_{r}^{+}$ and $w_{p}^{b-}$ are the weights of expected output r and unexpected output  p, respectively; $\varepsilon_{y}$ and $\varepsilon_{b}$ are parameters; φ is the output expansion ratio; $b_{pj}$ is the unexpected output  p of the decision-making unit j; $b_{pk}$ is the unexpected output  p of the decision-making unit k; and q is the total number of unexpected output  p.

Based on the efficiency index system of ESDE in China, this paper selects 6 types of input indicators, 1 item of expected output and 2 types of unexpected output in the non-guided, constant returns and unexpected output super-efficiency EBM model to measure the efficiency of China ‘s sustainable economic development.

#### 3.2.2. EBM-Malmquist Model

The above super-EBM model can only measure the static efficiency value of China’s sustainable economic development. In order to more comprehensively reflect the changes in the efficiency, the Malmquist index can be calculated to reflect the efficiency change rate of each decision-making unit. The Malmquist index was first proposed by Malmquist, and the Malmquist index uses the distance function to reflect the rate of change of each decision-making unit [52]. This paper uses the Malmquist index to reflect the changes in the ESDE of 30 provinces in China from 2008 to 2020. The mathematical expressions of the previous period t−1 and the current period t of the Malmquist index are shown in Formula (6) [53,54]:

(6) $$MI_{t-1,t}={\left[\frac{D_{t-1}(x_{t-1},y_{t-1})}{D_{t-1}(x_{t},y_{t})}\times \frac{D_{t}(x_{t-1},y_{t-1})}{D_{t}(x_{t},y_{t})}\right]}^{\frac{1}{2}}$$

where $x_{t-1}$ and $y_{t-1}$ represent the input vector and output vector of the previous period t−1, and $x_{t}$ and $y_{t}$ represent the input vector and output vector of the current period t. Variable $D_{t}(x_{t},y_{t})$ represents the distance between the decision-making unit in period t and the production frontier in period t−1, and the efficiency of a decision-making unit in period t is measured by constructing the production frontier with all decision-making units in period t−1. The value range of the Malmquist index is $MI_{t-1,t}\in (0,+\infty )$. Efficiency change of sustainable economic development in China depends on comparison with 1. If $MI_{t-1,t}>1$, it means that the efficiency of the current period is improved. If $MI_{t-1,t}<1$, it means that the efficiency of the current period has declined. If $MI_{t-1,t}=1$, it means that the efficiency of the current period remains unchanged.

### 3.3. Modified Gravity Model

The theory of economic gravity believes that there is an interactive relationship between regions within a certain range, and the construction of a spatial correlation matrix is a prerequisite for network analysis. Many scholars use a revised gravity model to measure the spatial correlation of research objects [38,55,56]. However, the connection between economies is different and unidirectional. When calculating the degree of connection between regional economies, it is necessary to take into account the economic scale, population and other related factors between economic regions.

The gravity model can be used to calculate the connection strength of the 30 provinces in China. The purpose of calculating the gravity matrix is to find the spatial correlation matrix of the ESDE. The gravity model is the basis of social network analysis, and the spatial correlation matrix in social network analysis can be converted from the correlation strength calculated by the gravity model. This paper draws on the approach of Zhang and Lu [57], and uses the following revised gravity model to calculate the spatial correlation strength among the 30 provinces in China.

(7) $$C_{ij}=K_{ij}\times \frac{\sqrt[{3}]{E_{i}G_{i}P_{i}}\times \sqrt[{3}]{E_{j}G_{j}P_{j}}}{D_{ij}^{2}/{\left(g_{i}-g_{j}\right)}^{2}},K_{ij}=\frac{E_{i}}{E_{i}+E_{j}}$$

where $C_{ij}$ represents the gravitational force between province i and province j, which represents the correlation strength of the ESDE; $K_{ij}$ represents the gravitational coefficient between province i and province j, representing the contribution of province i to the connection between province i and province j; $E_{i}$ and $E_{j}$ represent the ESDE of province i and province j; $G_{i}$ and $G_{j}$ represent the economic development level of province i and province j, measured by GDP; $P_{i}$ and $P_{j}$ represent the population of province i and province j; $D_{ij}$ represents the spherical distance between the capital city of province i and province j; $g_{i}$ and $g_{j}$ represent the per capita GDP of province i and province j; $g_{i}-g_{j}$ represents the economic distance of province i and province j.

Using the gravity model of Formula (7), the gravity matrix of the sustainable development efficiency of an inter-provincial economy can be constructed. The average value of the row values of the matrix $C_{ij}$ is used as the threshold value. If the gravity value is greater than the threshold, the value is 1, which means that the province in this row has an efficiency space overflow for the province in the column. Conversely, if it is lower than the threshold, the value is 0, indicating that there is no correlation effect between the two provinces. After the above conversion, the spatial correlation matrix I of ESDE can be obtained, which is actually a (0, 1)-matrix.

### 3.4. Social Network Analysis

Social Network Analysis (SNA) is an analysis method based on the perspective of “relationship”, including sociology, economics, geography and other disciplines [58]. It is mainly used to analyze the relationship structure and attributes of social networks, providing an “interactive” perspective and a global analysis by describing the relational schema with graph theory tools and algebraic modeling techniques [57]. Most multivariate statistical methods cannot be used to analyze relational data, and social network analysis can just make up for this limitation. The advantage of the social network analysis method is that it can accurately quantify various associations and avoid the limitations of “adjacent” or “similar” traditional spatial econometric analysis methods, thus providing a basis for the construction of some middle-level theory, and the test of empirical propositions provide quantitative tools. It is even possible to build a bridge between “macro and micro” (Liu and Jia, 2019). Therefore, this is also one of the important reasons why many experts and scholars apply social network analysis to economics, management and other fields.

Based on the spatial correlation matrix I calculated by the revised gravity model, this paper constructs a spatial correlation network based on the ESDE. Social network analysis is used to analyze the overall network and individual network characteristics of China’s sustainable economic development. Among them, the overall network characteristics are mainly quantified by four indicators: the network relationship coefficient, network density, network hierarchy, and network efficiency. Individual network characteristics use point-in-degree and point-out-degree to analyze the individual spillover effects and benefit relationship.

Social network analysis is one of the main contents of this paper. Based on the calculation of ESDE, the social network analysis method is used to analyze the cross-regional flow relationship of ESDE in 30 provinces in China. The connection of regions with such connections will form a geospatial network, and social network methods can visualize the strength of this connection.

## 4. Result Analysis

### 4.1. The Economic Sustainable Development Efficiency (ESDE)

#### 4.1.1. Analysis of Overall Efficiency

Based on the MaxDEA8Ultra software, the super-EBM model of unexpected output was used to calculate ESDE in China from 2008 to 2020 according to Formulas (4) and (5) (Table 2).

As shown in Table 2, 40% of China’s provinces had a ESDE greater than 1 in 2008; the proportion reached 56.67% in 2020, indicating that the overall level of sustainable economic development in China is on the rise.

**Figure 1:**
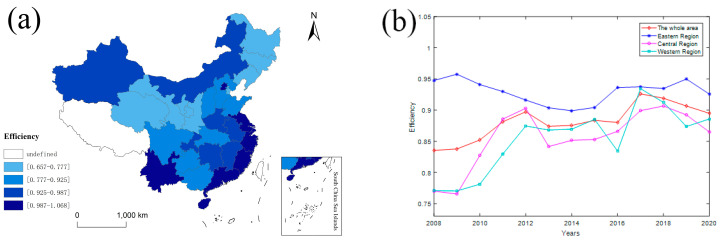
(**a**) The average level of ESDE in China; (**b**) the average level of ESDE in three regions. Data source: Table 2.

As shown on the right in Figure 1, the average of China’s ESDE showed an “M”-shaped fluctuation trend during 2008–2020. However, it showed an overall upward trend, with the average efficiency rising from 0.8354 in 2008 to 0.8818 in 2020. As shown on the left in Figure 1, it can be clearly seen that the ESDE of Beijing, Shanghai, Jiangsu, Zhejiang, Fujian, Guangdong, and Hainan is at a high level. The ESDE in Liaoning, Jilin, Heilongjiang, Shaanxi, Gansu, Qinghai, Ningxia and other provinces is at a low level.

**Figure 2:**
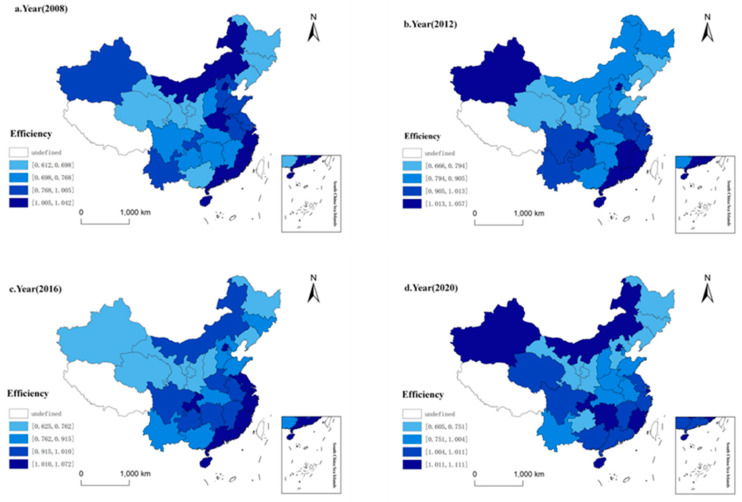
The spatial evolution pattern of efficiency of ESDE in China. Data source: Table 2.

As can be seen from Figure 2, Beijing, Shanghai, Fujian, Guangdong, Hainan, Zhejiang, Jiangsu maintain high efficiency. From 2008 to 2020, Henan and Hebei’s ESDE declined by two levels, and Guangxi, Qinghai, Hunan’s increased by two levels from 2008 to 2020. The areas where the efficiency level from 2008 to 2021 increased are Heilongjiang, Guangxi, Jiangxi, Sichuan, Guizhou, Chongqing, and Xinjiang. The areas where the efficiency level decreased are Inner Mongolia, Zhejiang, Henan, Hebei, Shandong, and Tianjin. The areas where the efficiency level from 2012 to 2016 increased are Tianjin, Jilin, Shandong, Inner Mongolia, Hunan, Jiangsu, and Zhejiang. The areas where the efficiency level decreased are Jiangxi, Xinjiang, Yunnan, Shanxi, Heilongjiang. The areas where the efficiency level from 2016 to 2020 increased are Shanxi, Qinghai, Xinjiang, Guangxi, Inner Mongolia, and Hunan. The areas where the efficiency level decreased are Jiangsu, Zhejiang, Guangdong, Anhui, Henan, Guizhou, Hebei, and Jilin.

#### 4.1.2. Analysis of Malmquist Index

Based on the efficiency results of the non-oriented, constant returns to scale, and unexpected output super-efficiency EBM-Malmquist model in the 30 provinces in China from 2008 to 2020, the MI index is decomposed into the EC index and the TC index. According to the Malmquist index, this paper analyzes the change trend of the ESDE of the 30 provinces (Table 3).

The MI index of each province in China fluctuated in different years. It can be seen from Table 3 that only Beijing’s ESDE increased in 2009, which is likely due to the impact of the global financial crisis, which seriously affected China’s economy. In 2010, except for Inner Mongolia and Shandong, the ESDE increased, but the MI index of Inner Mongolia and Shandong province increased significantly compared with 2009. In 2011, except for Beijing, Guangxi and Xinjiang, the ESDE in the other provinces declined. In 2020, the ESDE of most of the provinces in China declined, which may be due to the impact of COVID-19. As far as the province is concerned, during the period from 2008 to 2020, the ESDE in Beijing showed an increasing trend in most years, but declined in 2012, 2015 and 2020. The ESDE of Hebei Province only increased in 2010, 2016, 2018 and 2019. Anhui Province only increased in 2010, 2016, 2017 and 2018. According to the annual average MI index of the ESDE, only five provinces—Hebei, Shanxi, Liaoning, Heilongjiang, and Shandong—have an MI index less than one, indicating that the ESDE in most provinces in China is on the rise and is developing well. Provinces such as Hebei, Shanxi, Liaoning, Heilongjiang, and Shandong need to enhance their environmental governance capabilities and improve their ESDE.

### 4.2. Analysis of Regional Differences and Dynamic Evolution

#### 4.2.1. Estimation and Decomposition of Regional Differences in ESDE

In order to describe the level of regional differences and sources of regional differences in ESDE, this paper uses the Gini coefficient and its subgroup decomposition method proposed by Dagum (1997) to decompose the ESDE from 2008 to 2020. The Gini coefficient decomposition method solves the problem wherein the traditional Gini coefficient index does not satisfy the decomposability of subgroups—not only by taking the distribution of subgroup samples into account, but also by solving the problem of overlapping between sample data and the source of regional overall differences. The new Gini coefficient decomposition method can decompose the overall difference, see the source of the difference more clearly, and overcome the shortcomings of the traditional Gini coefficient and Theil coefficient (Liu et al., 2012; Li and Zhang, 2018). The formula of the Gini coefficient decomposition method is as follows:

(8) $$G=\frac{\sum_{j=1}^{k}\sum_{h=1}^{k}\sum_{i=1}^{n_{j}}\sum_{r=1}^{n_{h}}\left|y_{ji}-y_{hr}\right|}{2n^{2}y}$$

(9) $$G=G_{w}+G_{nb}+G_{t}$$

where k is the number of regions divided by China, k=3, which are the eastern region, central region and western region, respectively. Variables j and h are different regions in k regions, where j=1,⋯,k, h=1,⋯,k, j≠h. Variable n is the number of provinces in the research sample, n=30. Variable $y_{ji}(y_{hr})$ is the economic sustainable development efficiency of province i(r) in j(h) region, and y is the average value of the ESDE. The overall Gini coefficient can be decomposed into three parts: intra-regional gap contribution $G_{w}$, inter-regional gap contribution $G_{nb}$, and hypervariable density $G_{t}$. According to Formulas (8) and (9), the Gini coefficient and decomposition results of China’s ESDE are calculated, as shown in Table 4.

**Figure 3:**
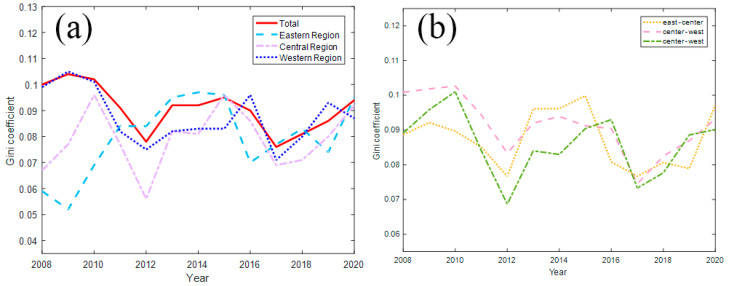
(**a**) Overall and intra-regional SDED differences; (**b**) inter-regional SDED differences. Data source: Table 4.

It can be seen from Table 4 and Figure 3 that the overall Gini coefficient of China’s ESDE increased by 4% in 2009, showed a slight increase, and declined from 2010 to 2012, with an average annual decline rate of 9%. After that, it rose in 2013, and then fluctuated slightly. During 2016–2017, it showed a downward trend, with a rate of decline of 10.41%, and reached its minimum value in 2017, and then showed an upward trend with a growth rate of 7.35% in 2018–2020, indicating that the overall regional differences in China’s ESDE fluctuate and have shown an expanding trend in recent years.

From the perspective of evolution, the differences in the ESDEs of the eastern region reached the minimum in 2009, and it showed an expanding trend during the period from 2010 to 2014, with an average annual growth rate of 13.93%, and reached the maximum in 2014. After 2015, regional differences showed a “W”-shaped fluctuation. The differences in the ESDEs of the central region showed a trend of substantial expansion during the period from 2008 to 2010 with a growth rate of 19.80%, and a decline rate of 23.53% during the period from 2010 to 2012. It reached the minimum in 2012, and then showed a fluctuating upward trend. During 2015–2016, there was a relatively large decline, with a decline rate of 14.47%. Then, the regional differences continued to widen during 2017–2020, with a growth rate of 12.59%. The trend of differences in the ESDEs of the western region during 2008–2015 is similar to the overall trend, with an average annual growth rate of 6.55% from 2013 to 2016, and fluctuates after 2016. In general, the difference between the whole area and the central region’s ESDE tends to expand after 2011, while the eastern and western regions show a fluctuating trend.

As shown on the right in Figure 3, China’s ESDE is uneven among regions, and the regional differences have shown a tendency to expand after 2017. The regional differences between the eastern and central regions show periodic characteristics, showing a “V”-shaped fluctuation trend during 2009–2015 and a weak “W”-shaped fluctuation trend during 2015–2020. In the sample, there is a “W”-shaped fluctuation trend. After 2009, the difference between the eastern region and the western region generally showed a fluctuating trend of “W”, showing a trend of “shrink–expand–shrink–expand”. After 2009, the regional differences between the central and western regions generally showed a “W” fluctuation trend, which was similar to the fluctuation trend of the differences between the eastern and western regions.

**Figure 4:**
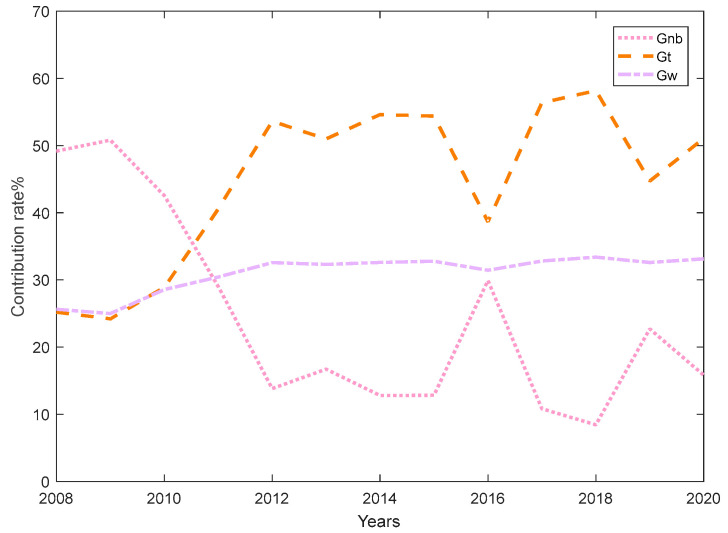
Differential contribution rate of ESDE from 2008 to 2020. Data source: Table 4.

Figure 4 reflects the contribution of inter-regional differences, hypervariable density and intra-regional differences to the overall differences. Prior to 2010, inter-regional differences contributed the most, and hypervariable density and intra-regional differences contributed almost equally to overall differences. After 2010, the contribution of the hypervariable density is the largest, and the hypervariable density is the main source of the overall difference. The contribution rate of the intra-regional difference remains basically unchanged, and the intra-regional contribution rate tends to expand. The contribution of intra-regional differences is significantly greater than that of inter-regional differences. Therefore, the key to solving the problem of unbalanced efficiency in China’s ESDE is to reduce the efficiency difference within the region.

#### 4.2.2. Dynamic Evolution of ESDE Regional Variation Distribution Based on 3D Kernel Density

Kernel density estimation is used to fit sample data by a smoothed peak function that describes the distribution pattern of random variables using a continuous density curve; it has the properties of robustness and weak model dependence. It has been widely used in dynamic evolution analysis.

**Figure 5:**
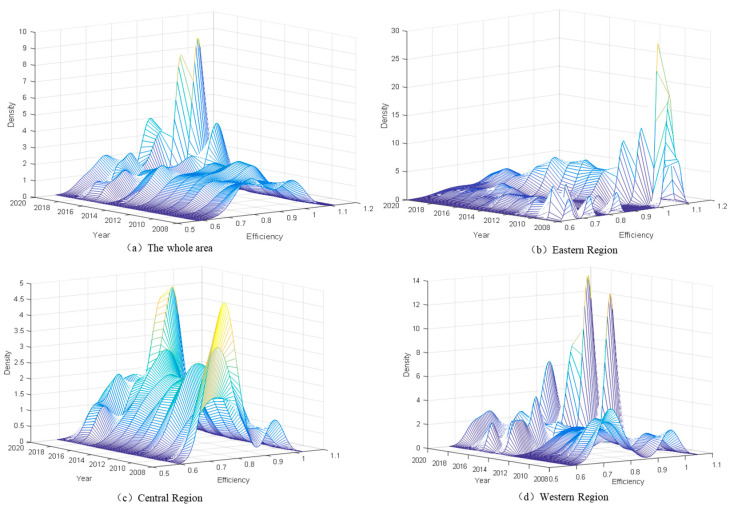
Three-dimensional Kernel density curves for (**a**) the whole area; (**b**) eastern region; (**c**) central region; (**d**) western region. Data source: obtained through Kernel density estimation according to the data in Table 2.

To describe the distributional features and dynamic evolution of the regional ESDE in China, we use Gaussian Kernel functions to estimate the density distribution pattern of the ESDE in China and to assess the dynamic evolution of the distribution via time dimension. Figure 5a–d illustrates the 3D Kernel density curves for China and the three major regions of eastern, central and western China, respectively.

Figure 5a indicates that between 2008 and 2020, the peak of the national regional ESDE shifts to the right and to then left, indicating that the overall ESDE shows a trend of a reversed U shape, which is consistent with the trend of the ESDE mean curve in previous discussions. During the sampling period, the overall curve shows a bimodal peak with a low side peak, indicating a weak polarization of ESDE. Years 2016, 2017 and 2018 appear to have higher peaks, indicating a convergence of ESDE towards high levels of development in these three years. The Kernel density estimation curves for China have an apparent bimodal pattern, with the side peaks appearing on the left side, pointing to a gradient effect and a polarization of ESDE within China.

As shown in Figure 5b, the bimodal pattern of the Kernel density curve of the ESDE in the eastern region is insignificant in the period from 2008 to 2020 and soon vanishes, evolving into a single-peaked pattern, indicating a weak polarization in the eastern region. The main peak of the ESDE in the eastern region shows a trend of “right shift–left shift–right shift–left shift”, indicative of a trend of “increase–decrease–increase–decrease” in the overall ESDE. The ESDE in the eastern region presents a fluctuating status and is at an overall high efficiency level. Meanwhile, the 2D density curve widens, showing a trend of widening differences between the eastern regions.

Figure 5c shows that the overall curve of ESDE in the central region shows a single-peaked pattern during the period from 2008 to 2020, indicating that there is no polarization. The curve shows a wide variation in the magnitude of the peak, with a trend of “high–low–high–low” and a trend of “right shift–left shift–right shift–left shift” in the position of the main peak, yet the variation is greater than in the eastern region, indicating that the overall ESDE follows an evolutionary trend of “increase–decrease–increase–decrease”. Within the sampling period, high peaks were observed in 2008, 2009 and 2018, indicating a clustering of ESDE towards high levels in these three years, which suggests an overall fluctuating trend in the level of sustainable economic development in the eastern region. Furthermore, the overall ESDE in the central region is significantly disparate, with a fluctuating level of development.

As shown in Figure 5d, the Kernel density curve in the western region appears to have multiple peaks, and the ESDE in the western region has a gradient effect, with multipolarity in some years. The main peak of the Kernel density curve in the western region shows a trend of “right shift–left shift–right shift–left shift”, indicating that the overall ESDE in the western region has an “increasing–decreasing–increasing–decreasing” trend. The Kernel density function center and curve in 2012 show a distinct rightward shifting trend, demonstrating a substantial increase in ESDE. Meanwhile, the curves in 2017 and 2018 show high peaks and high levels of average efficiency. Overall, ESDE has improved and grown significantly in recent years in the western region.

### 4.3. Analysis of Spatial Network Characteristics

#### 4.3.1. Overall Network Characteristics Analysis

In order to study the relationship among the level of sustainable economic development of the 30 provinces in China and the closeness of the connection among regions, this paper uses the social network analysis method to visualize the flow of sustainable economic development between different regions. This is convenient for targeting corresponding policies that should be implemented in accordance with the degree of regional correlation, thereby enhancing the level of sustainable economic development of the region through the flow between different provinces.

**Figure 6:**
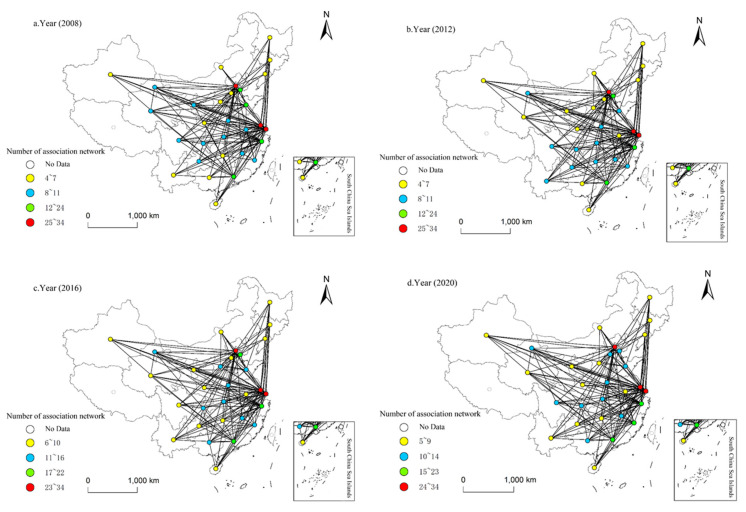
The spatial association network of regional economic growth in China. (**a**) Year 2008; (**b**) year 2012; (**c**) year 2016; (**d**) year 2020. Data source: calculated by the social network analysis according to the variables in Table 2.

The ESDE spatial correlation matrix is constructed based on the modified gravity model, and the spatial correlation network of China’s regional economic growth in 2008, 2012, 2016 and 2018 is visualized and mapped with ArcGIS (Figure 6).

Non-neighboring provinces break the traditional geographic constraints and produce complex cross-regional connections, with inter-provincial interaction and transmission existing through both proximity and jumping paths, resulting in a spatial structure characterized by polar nucleus fission and with multiple inter-provincial connections intertwined in a typical “spider web” structure. The average number of network associations between 2008 and 2020 reaches 191, and the network structure remains comparatively stable. Among the spatially associated networks, Beijing-Tianjin, the Yangtze River Delta region and Guangdong Province have the densest network associations, while the northeast, northwest and southwest regions have sparser network associations; the spatial association effect is higher in the eastern region than in the western region overall. The spatial association network remains relatively stable, with Beijing and Tianjin, the Yangtze River Delta and Guangzhou as the core circles, while the number of spatial associations in the central and western provinces is low, and the role of these provinces in the spatial association network is weak, showing an apparent “dense in the east and sparse in the west” pattern of regional differentiation.

**Figure 7:**
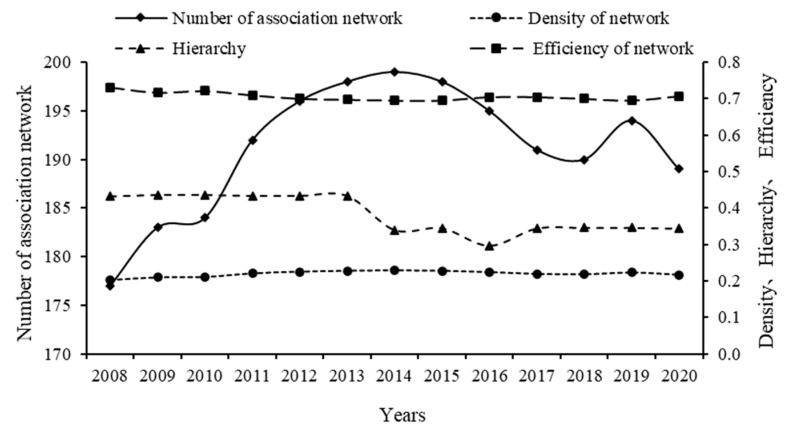
Overall structural trends of regional ESDE in China. Data source: Table 5.

Ucinet 6.0 software was used to produce the values of four indicators: number of association network, network density, hierarchy and network efficiency (Table 5) and to draw the plots (Figure 7).

Figure 7 and Table 5 show that the number of inter-provincial ESDE connections in China follows a “rising–fluctuating–declining” trend, network density and network efficiency remain relatively stable, network hierarchies show a “stable–declining–stable” trend, and network efficiency declines in general, indicating that the stability of the overall network structure has enhanced. Specifically, the number of network connections rose from 177 in 2008 to 199 in 2014, reaching a peak in 2014 with a stable network structure. This was followed by a fluctuating downward trend in the number of network connections between 2015 and 2019, though the decline was minor and within the scope of standard fluctuations. The number of network connections in 2020 decreased to 189 from 194 in 2019, most likely as a result of the COVID-19 pandemic outbreak in 2020 and a decrease in inter-provincial economic sustainability connections due to the increasing spatial liquidity of economic factor resources and a minor decrease in network connections under the influence of market regulation and government macro-control policies. Importantly, the network density value reflects the closeness of the connections between the regions in the network, and the maximum number of possible connections in the network is 870. During the study period, the actual number of connections and the network density value remains low and the closeness of the connected network has to be strengthened, indicating that the spatially connected exchange and spillover effects of the inter-provincial ESDE remains weak; the inter-provincial collaborative and balanced economic sustainable development has the potential to be improved, and the economic connections between regions need to be further enhanced. The network hierarchy remains stable at 0.4343 between 2008 and 2013, yet the network hierarchy declined from 0.4337 to 0.3404 in 2014, with a downward movement towards a ‘W’-shaped adjustment between 2014 and 2017, indicating that the spatially connected network hierarchy is less rigid and there is a possibility of spatial spillover between different regions. Spatial spillover is potentially possible between regions, and the interaction and interdependence between regions is growing. The network hierarchy moves to a stable level of approximately 0.3452 after 2017. The network efficiency level exceeds 0.6 across the study period; however, there is an overall downward trend from 0.7315 in 2008 to 0.7069 in 2020, implying that despite the existence of a higher number of spillover and redundant paths of interaction between regions, the cost of ESDE transmission and spillover between provinces is reduced and the paths of interaction move in a more reasonable and coordinated direction. The stability of the network is growing. Moreover, the overall network structure of ESDE is stable and continues to improve, yet has the potential to be improved.

#### 4.3.2. Individual Network Characteristics Analysis

To reveal the centrality characteristics of the individual networks in the spatial association network, three indicator values were calculated for each province: in-degree, out-degree and total number of associated connections (Table 6).

Table 6 shows the spillover–benefit relationship for the 30 provinces in China. Specifically, the in-degree represents the beneficial relationship and the out-degree represents the spillover relationship. The out-degree of Beijing, Tianjin, Shanghai, Jiangsu and Zhejiang is higher than the in-degree, indicating that Beijing, Tianjin and Yangtze River Delta have more spillover relationships than beneficiary relationships in the ESDE association network, forming a significant spillover effect on other regions; the out-degree of the northeast, northwest, southwest and central regions is less than the in-degree, suggesting that the these regions have fewer spillover relationships than beneficiary relationships, receiving more spillover from other regions in the association network and showing a significant beneficiary effect. The explanation for this is that the Beijing-Tianjin Urban Agglomeration and Yangtze River Delta regions are in the early stage of economic development mode transformation, focusing on environmental management and protection in the process of fast economic development. Under the pressure of national macro-control policies, especially under the promotion of the regional coordinated development strategy and the concept of sustainable development, capital and technology conducive to sustainable economic development have continued to spread to regions such as the central and western regions and the northeast, thus showing a strong spillover effect, while other regions have a markedly beneficial relationship.

## 5. Conclusions and Recommendation

### 5.1. Conclusions

At present, most of the literature on efficiency measurement uses human resources, capital and energy consumption as the input indicators of the EBM model. On this basis, this paper takes “social livelihood” and “environmental protection” as an input index. In order to prevent the potential bias of a single perspective on the total evaluation, six dimensions of input indicators—environment, resources, social livelihood, science and technology, capital, and labor—were designed. This study of the ESDE reflects the unity of human-centered attributes and natural environmental attributes, which fills the gap of previous studies. Based on the ESDE evaluation indicators, the EBM-Malmquist model of super-efficiency of non-expected output was constructed, which overcomes the problem of easy loss of information on the ratio between the target and actual values of inputs or outputs in the calculation and accurately measures the provincial ESDE in China. The Gini coefficient method was applied to analyze the regional differences and sources of differences in ESDE. Combined with the Gaussian density function estimation method, the density function of the distribution of regional differences and the dynamic evolution pattern are concluded. The spatial correlation matrix of ESDE is constructed based on the modified gravity model in combination with the variance analysis. The social network method was used to assess the overall ESDE network structural characteristics, individual network characteristics and spatial network spillover effects.

This study’s four main findings are as follows:

(1) China’s overall ESDE is on an upward trend. The ESDE in the eastern regions of Beijing, Shanghai, Jiangsu, Zhejiang and Guangdong are leading at high levels, while the ESDE in the central regions of Liaoning, Jilin and Heilongjiang are lagging behind. The western regions are mostly at a medium level of ESDE, the eastern regions are in the leading position, and the central and western regions are keeping up with the East, while ESDE in the Northeast is lagging behind.
(2) The overall regional differences in China’s ESDE have fluctuated and have tended to widen in recent years. On a national level, the ESDE exhibits a “gradient effect” and weak polarization. The ESDE in the eastern region does not appear to be polarized; however, there is a tendency for the differences to widen, and the ESDE in the central region is evidently disparate and fluctuates, while the ESDE in the western region appears to have a gradient effect with multi-polarization in some years.
(3) The regional ESDE has shown significant spatial unevenness, with Beijing-Tianjin, the Yangtze River Delta and Guangzhou as the core of the circle structure. The role of the central and western provinces in the spatial network is weak, showing an apparent pattern of geographical differentiation with high density in the east and low density in the west, and the spatial interaction and spillover effects are also weak. Furthermore, the ESDE’s inter-provincial transmission and spillover costs are decreasing, the interaction paths are moving towards a more reasonable and coordinated direction, and the stability of the network is improving. Overall, the network structure of ESDE is stable and continues to progress in a positive direction, though there is still considerable improvement potential.
(4) Beijing, Tianjin and the Yangtze River Delta have higher spillover relationships than beneficiary relationships in the correlation network, resulting in significant spillover effects on other regions; the node out-degrees of the northeast, northwest, southwest and central regions are less than the node in-degrees, as the spillover relationships in the above regions are lower than the beneficiary relationships; the regions receive more spillover effects from other regions in the correlation network and show significant beneficiary effects.

### 5.2. Recommendation

To promote the sustainable and balanced development of China’s economy, three policy recommendations are proposed on the basis of our research findings.

(1) The spillover relationship is low in central and western China, and their spillover effects need to be strengthened. The western region has greater advantages in terms of promoting clean energy compared to the eastern region; the rapid development of clean energy could significantly contribute to sustainable development. Moreover, it would be beneficial to strengthen capital and technology investment in the central and western regions, focus on clean energy development policies, and use clean energy development to enhance sustainable economic spatial spillover relations, thus supporting sustainable economic development.
(2) For economically less developed regions, the level of investment in environmental protection has to be enhanced while maintaining economic growth. These regions thus will need the support and supervision of government and national policies. Meanwhile, it would be beneficial to enhance the leading role of Beijing, Tianjin and the Yangtze River Delta in ESDE to strengthen the sustainable economic development of nearby regions.
(3) Strengthen the effectiveness of the policy impact of market regulation and government macro-control, accelerate the spatial movement of economic factor resources and improve the inter-provincial economic connections for sustainable development. Reduce channels for inter-regional spillover and redundant interactions to enhance the network structure of ESDE, lower the cost of ESDE transmission and spillover between provinces, enhance the hierarchy and stability of spatially connected networks, and comprehensively enhance China’s sustainable economic development.

In future research, a more refined division of input variables and output variables in the EBM-Malmquist model can be further studied so as to include most aspects of the connotation of ESDE and increase the measurement accuracy.

## Figures and Tables

**Table 1:** Efficiency Index System of China’s Regional Economic Sustainable Development.

Variables	The First Level	The Second Level	Unit	Weight
Input indicators	Capital	The stock of fixed asset investment	CNY 100 million	
	Environmental Governance	Local fiscal expenditure on environmental protection	CNY 100 million	0.4091
		Complete investment in industrial pollution control	CNY ten thousand	0.5909
	Social Security	Local fiscal social security and employment expenditures	CNY 100 million	0.3048
		local medical expenditure	CNY 100 million	0.3660
		Local fiscal education expenditure	CNY 100 million	0.3292
	Technology	Intramural expenditure on R&D	CNY ten thousand	0.5608
		Local fiscal science and technology expenditure	CNY 100 million	0.4392
	Resources	Total water supply	million tons	0.4456
		Urban Green Area	million hectares	0.3093
		Energy consumption	10,000 tce	0.2451
	Labor	Number of employees	ten thousand	
Output indicators	Expected Output	GDP	CNY 100 million	
	Undesired Output	COD	Tons	
		SO_2_	Tons	

**Table 2:** The ESDE of China’s 30 provinces from 2008 to 2020.

Region	ESDE					Region	ESDE				
	2008	2012	2016	2020	Average		2008	2012	2016	2020	Average
Beijing	1.0342	1.0559	1.0706	1.1101	1.0673	Hunan	0.7466	0.8969	1.0019	1.0183	0.9459
Tianjin	0.7585	0.6663	0.8032	0.8278	0.7342	Guangdong	1.0108	1.0147	1.022	1.0044	1.0134
Hebei	0.8444	0.8359	0.8047	0.6948	0.8153	Guangxi	0.6972	0.8851	0.7918	1.0065	0.8180
Shanxi	0.7653	0.8179	0.7092	0.7622	0.7825	Hainan	1.0407	1.0488	1.0499	1.0969	1.0600
Inner Mongolia	1.0074	0.8178	1.0090	1.0106	0.9547	Chongqing	0.7700	1.0143	1.0413	1.0155	0.9700
Liaoning	0.6946	0.696	0.7044	0.6049	0.6743	Sichuan	0.6997	0.9131	0.9215	1.0038	0.9050
Jilin	0.6548	0.7925	0.7883	0.7428	0.7355	Guizhou	0.7070	1.003	1.0055	0.7474	0.8895
Heilongjiang	0.6768	0.7971	0.6414	0.6142	0.6575	Yunnan	1.0038	1.009	0.9103	0.8195	0.9879
Shanghai	1.0165	1.0213	1.0176	1.0333	1.0233	Shaanxi	0.6122	0.7609	0.7556	0.7479	0.7264
Jiangsu	1.0045	1.0052	1.0152	1.0104	1.0111	Gansu	0.6780	0.742	0.6808	0.6944	0.7121
Zhejiang	1.0113	1.0035	1.0104	1.0066	1.0061	Qinghai	0.6516	0.7447	0.7371	1.0053	0.7753
Anhui	0.8191	1.0022	0.9188	1.0005	0.9533	Ningxia	0.6502	0.7141	0.6255	0.6554	0.6587
Fujian	1.0065	1.0169	1.0178	1.0243	1.0189	Xinjiang	1.0032	1.0160	0.6988	1.0368	0.9860
Jiangxi	0.7618	1.0241	1.0082	1.0070	0.9576	Eastern region	0.9474	0.9161	0.9363	0.9256	0.9294
Shandong	1.0000	0.7129	0.7831	0.7682	0.7992	Central region	0.7700	0.9028	0.8659	0.8649	0.8559
Henan	1.0048	1.0025	1.0047	1.0036	0.9708	Western region	0.7709	0.8746	0.8343	0.8857	0.8530
Hubei	0.7307	0.8894	0.8544	0.7704	0.8438	The whole area	0.8354	0.8973	0.8801	0.8948	0.8818

Data source: calculated by the super-EBM model according to the variables in Table 1.

**Table 3:** MI index, EC index and TC index of Chinese provinces.

Region	2009			2012			2016			2020		
	MI	EC	TC	MI	EC	TC	MI	EC	TC	MI	EC	TC
Beijing	1.0168	0.9989	1.0179	0.7158	0.9829	0.7283	1.0849	1.0151	1.0688	0.9948	0.9951	0.9997
Tianjin	0.9506	0.9612	0.9890	0.9452	0.9938	0.9510	1.2822	1.3115	0.9777	0.9098	0.9681	0.9398
Hebei	0.9704	1.1847	0.8191	0.9540	0.8349	1.1426	1.1507	1.1069	1.0396	0.8469	0.9515	0.8900
Shanxi	0.8627	0.9300	0.9276	0.9617	0.8171	1.1769	1.1124	0.9146	1.2163	0.8484	0.9660	0.8782
Inner Mongolia	0.7404	1.0001	0.7403	0.9989	1.0451	0.9558	1.1429	1.0033	1.1391	0.9906	0.9800	1.0109
Liaoning	0.9342	0.9935	0.9404	0.9879	1.0904	0.9061	1.1237	1.0112	1.1113	0.8059	0.8559	0.9415
Jilin	0.8959	0.9769	0.9171	0.9852	1.0821	0.9105	1.2162	1.1659	1.0432	0.9354	0.9810	0.9535
Heilongjiang	0.8799	0.9460	0.9301	1.0004	1.2143	0.8238	1.1256	1.0827	1.0396	0.9198	0.9539	0.9643
Shanghai	0.9994	1.0009	0.9985	0.9702	0.9967	0.9734	1.0879	0.9961	1.0921	1.0053	1.0028	1.0024
Jiangsu	0.9701	0.9994	0.9706	0.9063	0.9993	0.9070	1.1017	0.9999	1.1018	1.0054	0.9984	1.0070
Zhejiang	0.8935	0.9889	0.9035	0.9484	0.9976	0.9507	1.1537	1.0089	1.1435	1.0031	0.9981	1.0050
Anhui	0.9698	1.0277	0.9436	0.9835	0.9994	0.9841	1.0768	1.0308	1.0446	0.8201	0.9978	0.8219
Fujian	0.9893	1.0029	0.9865	0.8685	0.9986	0.8698	1.1171	0.9986	1.1187	0.9937	0.9984	0.9953
Jiangxi	0.9747	1.0244	0.9514	1.2541	1.0074	1.2449	1.0590	0.9989	1.0602	1.0030	1.0013	1.0017
Shandong	0.9681	1.0006	0.9675	0.9480	0.9991	0.9489	1.1467	1.0990	1.0434	0.8338	0.8723	0.9559
Henan	0.8351	0.9991	0.8359	0.9926	1.1497	0.8633	1.1351	1.0000	1.1351	0.9952	0.9944	1.0009
Hubei	0.9597	1.0192	0.9416	0.9841	1.0309	0.9546	1.0885	0.9791	1.1117	0.8095	0.8365	0.9677
Hunan	0.9500	1.0198	0.9315	0.9681	0.9524	1.0165	1.1034	1.0004	1.1030	1.0066	1.0087	0.9979
Guangdong	0.9941	0.9995	0.9946	0.8557	0.9955	0.8596	1.2144	1.0014	1.2126	0.9991	0.9932	1.0059
Guangxi	0.8808	1.0121	0.8702	0.9767	1.0317	0.9467	1.0630	0.9590	1.1085	0.9426	1.3057	0.7219
Hainan	0.8639	0.9981	0.8655	0.9668	0.9939	0.9727	0.9014	0.9860	0.9142	1.0789	1.0234	1.0542
Chongqing	0.9505	1.0154	0.9360	1.0091	1.0056	1.0035	1.3225	1.0238	1.2918	0.9810	0.9721	1.0091
Sichuan	0.9688	1.0182	0.9515	1.0360	1.1242	0.9215	1.1117	0.9206	1.2076	0.8367	0.9961	0.8400
Guizhou	0.9561	1.0129	0.9439	1.0529	1.2159	0.8660	1.3104	0.9892	1.3247	0.8646	0.9377	0.9220
Yunnan	0.9678	1.0023	0.9656	1.0068	0.9994	1.0073	1.0901	0.9076	1.2010	0.6957	0.8080	0.8611
Shaanxi	0.9358	0.9803	0.9546	1.0684	1.0977	0.9734	1.0999	1.0150	1.0837	0.8681	0.9692	0.8957
Gansu	0.9510	1.0184	0.9338	0.9882	1.0256	0.9635	1.1099	0.9703	1.1439	0.8753	0.9447	0.9266
Qinghai	0.8365	0.9283	0.9011	0.9722	1.0790	0.9010	1.1087	0.9820	1.1290	1.1512	1.1797	0.9758
Ningxia	0.9395	0.9946	0.9446	0.9890	1.0082	0.9809	1.0093	0.9346	1.0799	1.0458	1.1291	0.9262
Xinjiang	0.6847	0.9990	0.6854	0.7248	1.0022	0.7233	1.0198	0.6977	1.4617	1.0036	1.0312	0.9733
The whole area	0.9300	1.0032	0.9283	0.9650	1.0141	0.9583	1.1297	1.0425	1.0874	0.9247	0.9619	0.9609
Eastern region	0.9591	1.0117	0.9503	0.9152	0.9893	0.9282	1.1240	1.0486	1.0749	0.9524	0.9689	0.9815
Central region	0.9111	0.9890	0.9211	1.0231	1.0430	0.9940	1.1162	1.0246	1.0929	0.9045	0.9616	0.9412
Western region	0.8920	0.9983	0.8934	0.9839	1.0577	0.9312	1.1262	0.9457	1.1973	0.9323	1.0230	0.9148

Data source: calculated by the super-EBM-Malmquist model according to the variables in Table 1.

**Table 4:** Gini coefficient and its decomposition results from 2008 to 2020.

Year	G	$G_{w}$			$G_{nb}$			Contribution Rate (%)		
		East	Center	West	East–Center	Center–West	Center–West	$G_{nb}$	$G_{t}$	$G_{w}$
2008	0.1000	0.0590	0.0670	0.0990	0.0887	0.1009	0.0891	49.1700	25.2000	25.6300
2009	0.1040	0.0520	0.0770	0.1050	0.0922	0.1019	0.0959	50.8080	24.2030	24.9890
2010	0.1020	0.0690	0.0960	0.1010	0.0897	0.1027	0.1010	42.5430	28.8930	28.5630
2011	0.0910	0.0840	0.0770	0.0820	0.0852	0.0944	0.0841	29.0200	40.5560	30.4240
2012	0.0780	0.0840	0.0560	0.0750	0.0767	0.0833	0.0687	13.8190	53.6090	32.5720
2013	0.0920	0.0950	0.0820	0.0820	0.0961	0.0920	0.0840	16.7100	50.9820	32.3070
2014	0.0920	0.0970	0.0810	0.0830	0.0962	0.0939	0.0830	12.7920	54.6110	32.5970
2015	0.0950	0.0960	0.0960	0.0830	0.0999	0.0913	0.0904	12.8360	54.3850	32.7790
2016	0.0900	0.0700	0.0860	0.0960	0.0808	0.0904	0.0930	29.9030	38.6650	31.4310
2017	0.0760	0.0770	0.0690	0.0710	0.0767	0.0745	0.0733	10.8240	56.3640	32.8130
2018	0.0810	0.0830	0.0710	0.0800	0.0807	0.0825	0.0777	8.4360	58.1750	33.3890
2019	0.0860	0.0740	0.0800	0.0930	0.0789	0.0870	0.0886	22.6990	44.7110	32.5900
2020	0.0940	0.0950	0.0920	0.0870	0.0972	0.0932	0.0902	15.8240	51.0470	33.1290

Data source: calculated according to Formulas (8) and (9) based on the data in Table 2.

**Table 5:** Overall structural features of regional economic growth in China.

Year	2008	2009	2010	2011	2012	2013	2014	2015	2016	2017	2018	2019	2020
Number of association networks	177	183	184	192	196	198	199	198	195	191	190	194	189
Density of network	0.2034	0.2103	0.2115	0.2207	0.2253	0.2276	0.2287	0.2276	0.2241	0.2195	0.2184	0.223	0.2172
Hierarchy	0.432	0.4361	0.4361	0.4337	0.4337	0.4337	0.3404	0.3452	0.2974	0.3444	0.346	0.346	0.3444
Efficiency of network	0.7315	0.7167	0.7217	0.7094	0.6995	0.697	0.6946	0.6946	0.7044	0.7044	0.6995	0.6946	0.7069

Data source: calculated by the social network analysis according to the variables in Table 2.

**Table 6:** In/Out-degree and Number of Association Network of Inter-Provincial ESDE in China.

Region	Year 2008			Year 2012			Year 2016			Year 2020		
	In- Degree	Out- Degree	Number of Association Network	In- Degree	Out- Degree	Number of Association Network	In- Degree	Out- Degree	Number of Association Network	In- Degree	Out- Degree	Number of Association Network
Beijing	6	24	30	6	24	30	6	23	29	6	24	30
Tianjin	5	15	20	5	16	21	5	12	17	3	10	13
Hebei	4	3	7	5	4	9	5	4	9	5	5	10
Shanxi	5	2	7	5	3	8	6	5	11	5	2	7
Inner Mongolia	3	1	4	6	1	7	5	2	7	5	1	6
Liaoning	5	2	7	6	2	8	4	2	6	5	1	6
Jilin	6	1	7	6	1	7	6	0	6	5	0	5
Heilongjiang	6	1	7	7	1	8	7	0	7	7	0	7
Shanghai	7	27	34	8	27	35	7	27	34	7	27	34
Jiangsu	5	22	27	5	25	30	5	27	32	6	26	32
Zhejiang	4	20	24	4	19	23	4	18	22	4	19	23
Anhui	3	6	9	3	6	9	3	6	9	3	4	7
Fujian	7	1	8	9	6	15	7	6	13	8	10	18
Jiangxi	6	5	11	7	6	13	7	6	13	7	6	13
Shandong	7	11	18	6	9	15	7	9	16	5	9	14
Henan	6	5	11	6	9	15	6	8	14	6	6	12
Hubei	5	4	9	6	5	11	8	5	13	7	5	12
Hunan	5	2	7	7	3	10	7	3	10	7	2	9
Guangdong	10	14	24	10	11	21	11	10	21	10	10	20
Guangxi	5	1	6	6	2	8	7	4	11	7	3	10
Hainan	5	1	6	6	1	7	7	1	8	6	1	7
Chongqing	7	2	9	8	3	11	8	5	13	8	6	14
Sichuan	8	2	10	8	2	10	6	2	8	8	2	10
Guizhou	6	2	8	8	4	12	8	2	10	7	2	9
Yunnan	5	1	6	8	2	10	7	2	9	7	2	9
Shaanxi	7	0	7	7	1	8	6	1	7	5	1	6
Gansu	7	2	9	8	3	11	10	4	14	9	5	14
Qinghai	8	0	8	8	0	8	7	1	8	8	0	8
Ningxia	8	0	8	6	0	6	7	0	7	6	0	6
Xinjiang	6	0	6	6	0	6	6	0	6	7	0	7

Data source: Calculated by the social network analysis according to the variables in Table 2.

## Data Availability

The data used to support the findings of this study are available from the corresponding author upon request.

## References

[1] Khan I., Zakari A., Dagar V., Singh S. (2022). World energy trilemma and transformative energy developments as determinants of economic growth amid environmental sustainability. Energy Econ..

[2] Hake J.-F., Fischer W., Venghaus S., Weckenbrock C. (2015). The German Energiewende–history and status quo. Energy.

[3] Jaiswal K.K., Chowdhury C.R., Yadav D., Verma R., Dutta S., Jaiswal K.S., SelvaKumar K.K. (2022). Renewable and sustainable clean energy development and impact on social, economic, and environmental health. Energy Nexus.

[4] Wang S., Li Q., Fang C., Zhou C. (2016). The relationship between economic growth, energy consumption, and CO_2_ emissions: Empirical evidence from China. Sci. Total Environ..

[5] Alola A.A., Bekun F.V., Sarkodie S.A. (2019). Dynamic impact of trade policy, economic growth, fertility rate, renewable and non-renewable energy consumption on ecological footprint in Europe. Sci. Total Environ..

[6] Qiao Z., Meng X., Wu L. (2021). Forecasting carbon dioxide emissions in APEC member countries by a new cumulative grey model. Ecol. Indic..

[7] Venghaus S., Dieken S. (2019). From a few security indices to the FEW Security Index: Consistency in global food, energy and water security assessment. Sustain. Prod. Consum..

[8] Prasetyo P.E., Kistanti N.R. (2020). Human capital, institutional economics and entrepreneurship as a driver for quality & sustainable economic growth. Entrep. Sustain. Issues.

[9] Hoang T.C., Black M.C., Knuteson S.L., Roberts A.P. (2019). Environmental pollution, management, and sustainable development: Strategies for Vietnam and other developing countries. Environ. Manag..

[10] Pan S., Li B., Nie H. (2020). Research on the Path of China’s Sustainable Economic Development from the Perspective of Energy Constraints. Inq. Into Econ. Issues.

[11] Zhang J., Zhang N., Bai S. (2021). Assessing the carbon emission changing for sustainability and high-quality economic development. Environ. Technol. Innov..

[12] Liu H., Wang D., Shang J. (2020). From the Perspective of Green Low-Carbon Technological Progress:Can Green Credit Help Promote Sustainable Economic Development?. Jilin Univ. J. Soc. Sci. Ed..

[13] D’Adamo I., Gastaldi M., Morone P. (2022). Economic sustainable development goals: Assessments and perspectives in Europe. J. Clean. Prod..

[14] Atkinson G. (2009). Sustainable Development and Policy: A Brief Review of the Literature & Current Practice Final Report to the Government Economic Service Group on Sustainable Development. https://www.semanticscholar.org/paper/SUSTAINABLE-DEVELOPMENT-AND-POLICY-%3A-A-BRIEF-REVIEW-Atkinson/3ed85af99f97a36022e6dd60934c06e798a54d85.

[15] Stivers R.L. (1976). The Sustainable Society: Ethics and Economic Growth.

[16] Commission B. (1987). United Nations World Commission on Environment and Development. Our Common Future.

[17] Pezzey J.C. (1992). Economic Analysis of Sustainable Growth and Sustainable Development.

[18] Dailami M., Dhareshwar A., Kaufmann D., Kishor N., Lopez R., Thomas V., Wang Y. (2000). The Quality of Growth.

[19] Barro R.J. (2002). Quantity and Quality of Economic Growth.

[20] Brock W.A., Taylor M.S. (2005). Economic growth and the environment: A review of theory and empirics. Handb. Econ. Growth.

[21] Illge L., Schwarze R. (2009). A matter of opinion—How ecological and neoclassical environmental economists and think about sustainability and economics. Ecol. Econ..

[22] Bartelmus P. (2010). Use and usefulness of sustainability economics. Ecol. Econ..

[23] Wang Q., Huang R. (2021). The impact of COVID-19 pandemic on sustainable development goals—A survey. Environ. Res..

[24] Khan I., Hou F., Zakari A., Irfan M., Ahmad M. (2022). Links among energy intensity, non-linear financial development, and environmental sustainability: New evidence from Asia Pacific Economic Cooperation countries. J. Clean. Prod..

[25] Bai X., Wang K.-T., Tran T.K., Sadiq M., Trung L.M., Khudoykulov K. (2022). Measuring China’s green economic recovery and energy environment sustainability: Econometric analysis of sustainable development goals. Econ. Anal. Policy.

[26] Dabbous A., Tarhini A. (2021). Does sharing economy promote sustainable economic development and energy efficiency? Evidence from OECD countries. J. Innov. Knowl..

[27] Hosan S., Karmaker S.C., Rahman M.M., Chapman A.J., Saha B.B. (2022). Dynamic links among the demographic dividend, digitalization, energy intensity and sustainable economic growth: Empirical evidence from emerging economies. J. Clean. Prod..

[28] Winans K., Dlott F., Harris E., Dlott J. (2021). Sustainable value mapping and analysis methodology: Enabling stakeholder participation to develop localized indicators mapped to broader sustainable development goals. J. Clean. Prod..

[29] Qu J., Wang B., Liu X. (2022). A modified super-efficiency network data envelopment analysis: Assessing regional sustainability performance in China. Socio-Econ. Plan. Sci..

[30] Tao X., Wang P., Zhu B. (2016). Provincial green economic efficiency of China: A non-separable input–output SBM approach. Appl. Energy.

[31] Zhao P.-j., Zeng L.-e., Lu H.-y., Zhou Y., Hu H.-y., Wei X.-Y. (2020). Green economic efficiency and its influencing factors in China from 2008 to 2017: Based on the super-SBM model with undesirable outputs and spatial Dubin model. Sci. Total Environ..

[32] Wen X., Yao S., Sauer J. (2022). Evaluation of sustainable development considering natural conditions: A parametric slacks-based measure of efficiency approach. J. Clean. Prod..

[33] Swain R.B., Ranganathan S. (2021). Modeling interlinkages between sustainable development goals using network analysis. World Dev..

[34] Wang Z., Jia X. (2022). Analysis of energy consumption structure on CO_2_ emission and economic sustainable growth. Energy Rep..

[35] Ren Y., Fang C., Li G. (2020). Spatiotemporal characteristics and influential factors of eco-efficiency in Chinese prefecture-level cities: A spatial panel econometric analysis. J. Clean. Prod..

[36] Tone K., Tsutsui M. (2010). An epsilon-based measure of efficiency in DEA–a third pole of technical efficiency. Eur. J. Oper. Res..

[37] Che L., Bai Y., Zhou L., Wang F., Ji X., Qiao F. (2018). Spatial pattern and spillover effects of green development efficiency in China. Sci. Geogr. Sin..

[38] Zhao L., Cao N., Han Z., Gao X. (2021). Spatial correlation network and influencing factors of green economic efficiency in China. Resour. Sci..

[39] Ren J., Ma Y. (2020). Research progress and prospects of green development from the perspective of geography. J. Geogr. Cartogr..

[40] Chen M., Liu W., Wang S., Liu Y. (2020). Spatial pattern and temporal trend of urban ecological efficiency in the Yangtze River Economic Belt. Res. Sci..

[41] Zhang H., Geng Z., Yin R., Zhang W. (2020). Regional differences and convergence tendency of green development competitiveness in China. J. Clean. Prod..

[42] Wang Z., Wang X., Liang L. (2019). Green economic efficiency in the Yangtze River Delta: Spatiotemporal evolution and influencing factors. Ecosyst. Health Sustain..

[43] Sun C., Tong Y., Zou W. (2018). The evolution and a temporal-spatial difference analysis of green development in China. Sustain. Cities Soc..

[44] Liu H., Jia W. (2019). Spatial network correlation and convergence test of regional economic growth in China. Sci. Geogr. Sin..

[45] Liu S., Xiao Q. (2021). An empirical analysis on spatial correlation investigation of industrial carbon emissions using SNA-ICE model. Energy.

[46] Zhang R., Tai H., Cheng K., Zhu Y., Hou J. (2022). Carbon emission efficiency network formation mechanism and spatial correlation complexity analysis: Taking the Yangtze River Economic Belt as an example. Sci. Total Environ..

[47] Shen W., Liang H., Dong L., Ren J., Wang G. (2021). Synergistic CO_2_ reduction effects in Chinese urban agglomerations: Perspectives from social network analysis. Sci. Total Environ..

[48] Ding L., Wu M., Jiao Z., Nie Y. (2022). The positive role of trade openness in industrial green total factor productivity—Provincial evidence from China. Environ. Sci. Pollut. Res..

[49] Andersen P., Petersen N.C. (1993). A procedure for ranking efficient units in data envelopment analysis. Manag. Sci..

[50] Tone K. (2002). A strange case of the cost and allocative efficiencies in DEA. J. Oper. Res. Soc..

[51] Wang K., Liu Y., Shi L., Liu L., Meng X., Yang B. (2017). Yangtze River Economic Zone spatial and temporal disparities in industrial green water resource efficiency and influencing factors based on two-step analysis of EBM-Tobit Model. Resour. Sci..

[52] Malmquist S. (1953). Index numbers and indifference surfaces. Trab. de Estadística.

[53] Yun-jun Z., Yun T. (2021). An Analysis of the Evolution of and Difference in Tourism Efficiency of Key Tourism Cities in China—Based on the Super-Efficiency EBM Model. J. Southwest Univ..

[54] Huang R., Li X. (2021). The Mechanism and Path for Digital Technology to Improve the Efficiency of China’s Tourism Industry. Contemp. Econ. Res..

[55] Wei L. (2018). Influence of Urban Agglomeration Economic Cooperation on Regional Coordinated Development: Based on Comparison Between Beijing-Tianjin-Hebei and Shanghai-Jiangsu-Zhejiang. Sci. Geogr. Sin..

[56] Liu H., Jia W. (2019). Convergence test and coordinated development of China’s regional economic growth under different spatial network correlations. Nankai Econ. Stud..

[57] Zhang D., Lu Y. (2017). Study on the Spatial Correlation and Explanation of Carbon Emission in China—Based on Social Network Analysis. Soft Sci..

[58] Scott J. (2017). Social Network Analysis.

